# Supplementary material for: Development of a Droplet Digital PCR to Monitor SARS-CoV-2 Omicron Variant BA.2 in Wastewater Samples
Source: Microorganisms. 2023 Mar 12;11(3):729. doi: 10.3390/microorganisms11030729 (PMC10059707; doi:10.3390/microorganisms11030729)
Supplement: Supplementary file 1 [file microorganisms-11-00729-s001.zip › Table S2.pdf]

Table S2. List of DNA and RNA controls that were used for the *in vitro* specificity evaluation.

Kingdom	Genus	Species	Strain number	Collection / Company
**Animalia**	*Homo*	*sapiens*	/	Promega, G3041
**Plantae**	*Zea*	*mays*	/	ERM-BF413ak
**Bacteria**	*Bacillus*	*subtilis*	SI0005	Sciensano collection, Brussels, Belgium
	Escherichia	*coli*	MB1068	BCCM collection, Brussels, Belgium
**Fungi**	*Aspergillus*	*acidus*	26285	BCCM collection, Brussels, Belgium
	Candida	*cylindracea*	041387	BCCM collection, Brussels, Belgium
	**Family**		**Species**	**I19 Mutant**
**Viruses**	*Picornaviridae*		Rhinovirus B	Vircell, Granada, Spain - MBC091
	Reoviridae		Rotavirus	Vircell, Granada, Spain - MBC026
	Orthomyxoviridae		Influenza A (H1N1)	Vircell, Granada, Spain - MBC082
	Orthomyxoviridae		Influenza A (H3)	Vircell, Granada, Spain - MBC029
	Orthomyxoviridae		Influenza B	Vircell, Granada, Spain - MBC030
	Adenoviridae		Adenovirus	Vircell, Granada, Spain - MBC001
	Picornaviridae		Enterovirus D68	Vircell, Granada, Spain - MBC125
	Caliciviridae		Norovirus	Vircell, Granada, Spain - MBC111
	Pneumoviridae		RSV A	Vircell, Granada, Spain - MBC041
	Coronaviridae		SARS-CoV	Vircell, Granada, Spain - MBC136-R
	Coronaviridae		MERS-CoV	Vircell, Granada, Spain - MBC132
	Coronaviridae		Corona OC43	Vircell, Granada, Spain - MBC135-R
	Coronaviridae		Coronavirus control	- Vircell, Granada, Spain - MBC090-R
	Coronaviridae		SARS-CoV-2 WT	Vircell, Granada, Spain - MBC137
	Coronaviridae		SARS-CoV-2 B.1.1.7	Vircell, Granada, Spain - MBC138-R
	Coronaviridae		SARS-CoV-2 B.1.351	Vircell, Granada, Spain - MBC139-R
	Coronaviridae		SARS-CoV-2 P.1	Vircell, Granada, Spain - MBC140-R
	Coronaviridae		SARS-CoV-2 B.1.617.2	Vircell, Granada, Spain - MBC141-R
	Coronaviridae		SARS-CoV-2 BA.1	Vircell, Granada, Spain - MBC143-R
	Coronaviridae		SARS-CoV-2 BA.2	Vircell, Granada, Spain- MBC145-R
